# Genotype‐Dependent Variations in Egg Quality and Nutritional Composition of Thermotolerant Chickens Under Hot‐Climate Conditions

**DOI:** 10.1002/fsn3.72057

**Published:** 2026-06-29

**Authors:** Fatiha Soltani, Zineb Bengharbi, Djilali Benabdelmoumene, Said Dahmouni, Muhammad Waqar, Wasim S. M. Qadi, Ahmed mediani, Golam Sagir Ahammad

**Affiliations:** ^1^ Applied Animal Physiology Lab Abdelhamid Ibn Badis University Mostaganem Algeria; ^2^ Food Technology and Innovation Research Center of Excellence, School of Agricultural Technology and Food Industry Walailak University, Thai Buri Tha Sala Nakhon Si Thammarat Thailand; ^3^ Institute of Systems Biology (INBIOSIS) Universiti Kebangsaan Malaysia Bangi Selangor Malaysia; ^4^ Bangladesh Agricultural University Mymensingh Bangladesh; ^5^ Bangladesh Institute of Research and Training on Applied Nutrition (BIRTAN) Araihazar Narayanganj Bangladesh

**Keywords:** amino acid composition, egg quality, fatty acid profile, Fayoumi, Frizzled, hot‐climate production, mineral content, Naked Neck

## Abstract

Egg quality and nutritional composition may vary among thermotolerant chicken genotypes reared under hot‐climate conditions, with potential implications for nutritional value and climate‐resilient poultry production. This study compared egg morphometric traits, amino acid composition, yolk fatty acid profile, and mineral content in Fayoumi, Naked Neck, and Frizzled laying hens maintained under the same general feeding and management conditions during the summer period. Naked Neck hens produced the heaviest eggs (55.29 ± 4.11 g) compared with Frizzled (50.18 ± 3.71 g) and Fayoumi hens (48.92 ± 3.85 g), and also showed the highest albumen height (5.81 ± 0.14 mm) and Haugh unit (83.39 ± 1.55). Total protein content ranged from 12.80 ± 0.60 to 13.28 ± 0.11 g/100 g edible portion, whereas individual amino acids displayed clearer variation among genotypes. Fayoumi eggs showed the highest methionine concentration (320.40 ± 20.10 mg/100 g dry matter), Naked Neck eggs the highest leucine level (636.27 ± 82.93 mg/100 g dry matter), and Frizzled eggs the highest histidine content (211.55 ± 6.98 mg/100 g dry matter). Yolk lipid composition also differed among groups: Fayoumi eggs had the highest total lipid (8.92% ± 0.64%) and PUFA proportions (19.10% ± 0.95%), Naked Neck eggs showed the highest MUFA (49.61% ± 1.55%) and total ω3 contents (3.62% ± 0.16%), including the highest DHA level (1.26% ± 0.05%), whereas Frizzled eggs exhibited the lowest ω6/ω3 ratio (3.76 ± 1.48). Mineral composition was more conserved, with sodium, calcium, phosphorus, and potassium remaining comparable across genotypes, whereas iron content differed significantly and was highest in Naked Neck eggs (1.30 ± 0.19 mg/egg) compared with Fayoumi (1.24 ± 0.15 mg/egg) and Frizzled eggs (1.10 ± 0.08 mg/egg). The three genotypes exhibited distinct compositional profiles in the egg matrix, involving morphometric traits, amino acid distribution, lipid fractions, and iron deposition under the present experimental conditions.

## Graphical abstract

Hot‐climate conditions revealed clear genotype‐dependent differences in egg quality and nutritional composition among Fayoumi, Naked Neck, and Frizzled hens. These thermotolerant genotypes showed distinct amino acid, lipid, omega‐3, and iron profiles, supporting climate‐resilient poultry production.

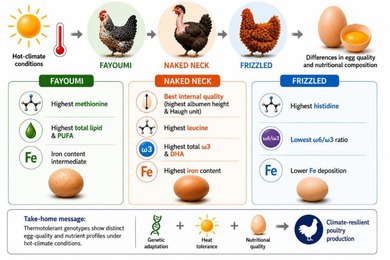

## 1 Introduction

Eggs are nutritionally dense animal‐derived foods that provide highly digestible proteins, indispensable amino acids, lipids, vitamins, choline, carotenoids, and essential minerals within a compact biological matrix (Waqar et al. 2025). Their affordability and high nutrient density make them important for food and nutritional security, particularly in tropical and subtropical regions where heat stress, water scarcity, limited land availability, and restricted dietary diversity constrain animal production systems (David et al. 2024; Wanapat et al. 2024). In these environments, sustainable egg production depends strongly on the ability of laying hens to maintain physiological and productive performance under elevated ambient temperatures (Kennedy et al. 2022; Soares et al. 2022; Olaniyan et al. 2024; Dessie et al. 2025). Indigenous and thermotolerant chicken genotypes are therefore valuable genetic resources because they combine adaptability, disease resistance, and physiological robustness under challenging production conditions. Among them, Naked Neck, Frizzled, and Fayoumi chickens have attracted increasing attention for their potential contribution to heat‐resilient poultry production (Fathi et al. 2024; Taye et al. 2024; Adomako and Asamoah 2025; Shafiq et al. 2025; Zaid et al. 2025).

Thermotolerant phenotypes may influence egg quality through genotype‐dependent differences in heat dissipation, metabolic allocation, and reproductive physiology. The Naked Neck genotype, characterized by reduced feather coverage, improves sensible heat loss and may support productive performance under hot conditions (Fernandes et al. 2023; Hemanth et al. 2024; Zineb et al. 2024; Juárez et al. 2025). Similarly, the Frizzle phenotype, defined by structurally modified feathers that enhance convective heat exchange, has been associated with improved thermal tolerance in warm climates (Nawaz et al. 2024; Adomako and Asamoah 2025). Fayoumi chickens are also recognized for resilience and disease resistance, making them relevant to extensive and semi‐intensive poultry systems. However, egg quality is not determined by thermal adaptation alone; it reflects the interaction between genetic background, reproductive status, nutrition, and environmental conditions. Genotype‐related differences in shell traits, albumen quality, yolk proportion, and freshness indicators have been linked to reproductive organ development, endocrine regulation, calcium metabolism, and egg structural integrity (Alam et al. 2021; Liu et al. 2024; Menchetti et al. 2024; Rho and Cho 2024; Al‐Khalaifah et al. 2025; Gao et al. 2025).

From a nutritional and biochemical perspective, egg quality extends beyond physical traits to the biochemical organization of the egg as a complex biological matrix (Ruangprom et al. 2025). Genotype can influence nutrient partitioning into yolk and albumen, thereby affecting amino acid composition, lipid architecture, and mineral deposition. Breed‐dependent differences in egg amino acid profiles suggest that genetic variation may modify protein deposition and the distribution of essential and non‐essential amino acids within egg components (Goto et al. 2021; Gyawali and Goto 2025). Similarly, hen genotype can modulate yolk lipid composition, including the proportions of saturated, monounsaturated, and polyunsaturated fatty acids, with direct relevance to nutritional quality and human health (Hejdysz et al. 2024; Oketch and Heo 2025). Long‐chain omega‐3 fatty acids are of particular interest because of their roles in cardiovascular, neurophysiological, and anti‐inflammatory functions, while minerals such as iron, zinc, selenium, calcium, and phosphorus contribute to the micronutrient value of eggs, especially in diets where these elements may be limiting (Akinwumi et al. 2025; Caffa et al. 2025).

These compositional traits are governed by coordinated metabolic processes in the laying hen. Yolk formation depends mainly on hepatic lipid synthesis and the transport of very low‐density lipoproteins and vitellogenin to the ovary, whereas albumen composition reflects regulated amino acid partitioning and protein secretion pathways that may vary according to genotype and physiological status (Attia et al. 2022; Van Eck et al. 2023; Usturoi et al. 2025). Eggshell mineralization further requires precise endocrine and metabolic control of calcium and phosphorus homeostasis (Hincke et al. 2019). Under heat stress, genotype‐dependent differences in thermoregulation, oxidative balance, and metabolic prioritization may influence not only egg size and internal quality but also amino acid balance, fatty acid composition, and mineral deposition (Akbarian et al. 2016; Attia et al. 2018). Such regulation is central to understanding compositional variation in animal‐derived foods before post‐harvest handling or processing (Berry et al. 2017; Wegner et al. 2023; Hejdysz et al. 2024).

Despite this relevance, integrated comparative data on egg morphometric traits, amino acid composition, yolk fatty acid profile, and mineral content in Fayoumi, Naked Neck, and Frizzled hens reared under hot‐climate conditions remain limited. Most available studies have focused on commercial strains, isolated quality traits, or dietary enrichment strategies rather than simultaneous evaluation of physical and biochemical attributes across thermotolerant genotypes maintained under comparable production conditions. Therefore, the present study aimed to evaluate genotype‐associated differences in egg quality and nutritional composition among Fayoumi, Naked Neck, and Frizzled laying hens reared under the same general feeding and management conditions during the summer period. By combining egg morphometric traits with targeted amino acid profiling, fatty acid composition, and mineral determination, this work clarifies how thermotolerant chicken genotypes differ in the nutritional architecture of eggs. Because each genotype was housed in a separate non‐replicated section, the findings are interpreted as genotype‐associated patterns under the present production setting rather than as definitive genetic causality. The study hypothesized that Fayoumi, Naked Neck, and Frizzled hens would exhibit distinct patterns of egg physical quality and nutritional composition under hot‐climate conditions, supporting the valorization of indigenous poultry resources for climate‐resilient and nutrition‐oriented egg production.

## 2 Materials and Methods

### 2.1 Egg Selection and Sample Preparation

The study was conducted from June to August under summer conditions in northern Algeria. Eggs were collected from Fayoumi, Naked Neck, and Frizzled laying hens at 30 weeks of age, corresponding to the stabilized laying period. For each genotype, 20 hens were included, and five eggs per hen were collected over five consecutive laying days, yielding a total of 100 eggs per genotype. This sampling strategy was adopted to compare egg characteristics among genetically distinct hen groups at the same physiological stage of lay while limiting temporal dispersion across the collection period.

All birds were maintained at a single breeder farm under the same general feeding and management program. Each genotype was housed in a separate section of the same poultry house, with comparable stocking density, lighting schedule, ventilation, and ad libitum access to feed and water. Under these standardized conditions, genotype represented the primary biological source of variation among the experimental groups.

All hens received the same maize–soybean meal‐based layer diet formulated to meet the nutrient requirements of laying hens, without lipid enrichment or functional additives; ingredient composition, calculated nutrient content, and major fatty acid profile of the experimental diet are presented in Table 1. Accordingly, major non‐genetic factors known to influence egg quality and composition, including age, diet formulation, management routine, sampling period, and post‐collection handling, were kept as constant as possible across the three genotypes.

**TABLE 1:** Ingredient composition, calculated nutrient content, and major fatty acid profile of the experimental diet.

Item	Content
Ingredients (%)	
Maize	55
Soybean meal (44% CP)	22
Wheat bran	7
Vegetable oil	2.5
Limestone	9
Dicalcium phosphate	1.6
Salt (NaCl)	0.3
Vitamin–mineral premix^a^	0.5
DL‐methionine	0.1
Calculated nutrient composition	
Metabolizable energy (MJ/kg)	11.5
Crude protein (%)	16.5
Ether extract (%)	4.5
Crude fiber (%)	3.8
Calcium (%)	3.6
Available phosphorus (%)	0.45
Lysine (%)	0.85
Methionine (%)	0.38
Methionine + cysteine (%)	0.65
Sodium (%)	0.16
Major fatty acids (% of total fatty acids)	
Palmitic acid (C16:0)	18
Stearic acid (C18:0)	4
Oleic acid (C18:1 n‐9)	30
Linoleic acid (C18:2 n‐6)	42

^a^ Vitamin–mineral premix supplied per kg of diet: Vitamin A, 10,000 IU; Vitamin D_3_, 2000 IU; Vitamin E, 30 mg; Vitamin K_3_, 2 mg; Vitamin B_1_, 2 mg; Vitamin B_2_, 5 mg; Vitamin B_6_, 3 mg; Vitamin B_12_, 0.01 mg; niacin, 30 mg; folic acid, 1 mg; biotin, 0.05 mg; Fe, 50 mg; Zn, 60 mg; Mn, 70 mg; Cu, 8 mg; I, 1 mg; Se, 0.3 mg.

Birds were sampled from pre‐existing genotype groups maintained by the breeder, and no experimental random allocation of hens to housing sections was performed specifically for this study. Eggs were collected within 24 h of oviposition, visually inspected, and only eggs free of cracks, shell defects, or obvious abnormalities were retained for analysis. Each egg was individually labeled according to genotype and hen identity, then transported to the laboratory under insulated conditions. Samples were stored at 4°C for no longer than 48 h before analysis. Prior to morphometric measurements, eggs were equilibrated to room temperature to minimize temperature‐related variability.

All eggs were first subjected to morphometric evaluation. Because subsequent amino acid, fatty acid, and mineral determinations were destructive, subsets of eggs were allocated to each analytical endpoint. For chemical analyses, the individual egg was considered the biological replicate, whereas analytical duplicates, when performed, were averaged before statistical analysis. The exact number of eggs analyzed for each analytical endpoint is reported in the corresponding tables.

### 2.2 Egg Morphometric Measurements

Egg morphometric traits were measured on all eggs prior to breaking using standardized procedures commonly applied in egg quality assessment (Franco et al. 2020; Sokołowicz et al. 2019). To minimize temperature‐related variation, eggs were equilibrated to room temperature (22°C–24°C) before analysis. All measurements were performed on individual eggs, and no averaging was conducted prior to statistical analysis. Egg weight was determined using a digital analytical balance (±0.01 g). Egg length (major axis) and width (minor axis) were measured using a digital caliper (±0.01 mm), and the egg shape index was calculated as egg width divided by egg length × 100 (Kayadan and Uzun 2023). After breaking, yolk and albumen were carefully separated and weighed individually. Albumen height was measured in the thick albumen region using a digital micrometer, and Haugh units were calculated according to standard equations to assess internal egg quality. Eggshells were rinsed with distilled water to remove adhering albumen, air‐dried at room temperature, and weighed. Shell thickness was measured using a digital micrometer (±0.001 mm) at three standardized locations (air cell, equator, and sharp end), and the mean value was calculated (Adeoye et al. 2023; Kamanlı et al. 2021). Yolk, albumen, and shell proportions were expressed as percentages of total egg weight to enable standardized comparison of egg component distribution among genotypes. Because multiple eggs were collected from the same hens over consecutive laying days, morphometric measurements were treated as repeated observations nested within hens and collection days.

### 2.3 Amino Acid Analysis

The amino acid composition of egg yolk and albumen was determined using acid hydrolysis followed by chromatographic separation, according to standardized procedures widely applied in food and egg protein analysis. Yolk and albumen were analyzed separately. For each genotype, amino acid analyses were conducted on individual eggs, and the individual egg constituted the biological replicate, while analytical duplicates were performed to assess repeatability. Samples were homogenized, freeze‐dried, and accurately weighed prior to hydrolysis. Proteins were hydrolyzed using 6 N hydrochloric acid (HCl) at 110°C for 24 h in sealed tubes under a nitrogen atmosphere to limit oxidative degradation. After hydrolysis, samples were cooled, filtered, neutralized, and subjected to chromatographic analysis. Amino acids were quantified by high‐performance liquid chromatography (HPLC) with post‐column derivatization and UV detection. Identification and quantification were achieved by comparison with authenticated external amino acid standards. Amino acid contents are expressed as mg/100 g dry matter. Tryptophan was not quantified in the present study, as it is degraded during acid hydrolysis, and was therefore excluded from all tables, statistical analyses, and multivariate models. In addition, performic acid oxidation was not applied prior to hydrolysis; consequently, sulfur‐containing amino acids (methionine and cysteine) may be underestimated. These values are therefore considered conservative estimates and were interpreted with caution. Accordingly, conclusions regarding essential amino acid balance focused on relative differences among genotypes analyzed under identical analytical conditions rather than on absolute nutritional adequacy. This approach allowed standardized comparison of amino acid distribution between yolk and albumen across the evaluated genotypes (Restrepo Osorio 2019; Alagawany et al. 2021; Ariza et al. 2021; Goto et al. 2021).

### 2.4 Fatty Acid Analysis

Total lipids were extracted separately from egg yolk and albumen using the method of Folch et al. (1957) with a chloroform–methanol mixture (2:1, v/v). Samples were homogenized, filtered, and washed with aqueous sodium chloride to induce phase separation. The organic phase was recovered and evaporated under a gentle nitrogen stream. Fatty acid methyl esters (FAMEs) were prepared by base‐catalyzed transesterification using methanolic potassium hydroxide according to Joseph and Ackman (1992). FAMEs were extracted with hexane, concentrated under nitrogen, and transferred to chromatographic vials for analysis. FAMEs were analyzed by gas chromatography with flame ionization detection (GC‐FID). Individual fatty acids were identified by comparison of retention times with authenticated FAME standards. Quantification was performed by area normalization, and fatty acid composition was expressed as relative proportions (% of total identified fatty acids). Because internal standards and response‐factor corrections were not applied, the reported values were interpreted as relative compositional profiles rather than absolute fatty acid concentrations. Because fatty acid proportions constitute compositional data, statistical analyses were performed on centered log‐ratio (CLR) transformed values to avoid spurious correlations inherent to percentage data. In addition, nutritionally relevant indices, including PUFA/SFA and n‐6/n‐3 ratios, were calculated to support biologically meaningful interpretation. Egg albumen contains very low lipid concentrations; therefore, fatty acid profiles obtained from albumen were considered qualitative. To minimize potential yolk carryover, yolk and albumen were separated carefully, visually inspected for contamination, and processed using dedicated tools. Solvent blanks were included among analytical runs. Albumen fatty acid data close to the analytical detection limit were interpreted with caution and were not used for absolute quantitative inference. Fatty acid composition was also summarized into saturated, monounsaturated, and polyunsaturated fatty acid classes to facilitate comparison of genotype‐associated lipid patterns under identical analytical conditions.

### 2.5 Mineral Analysis

Mineral composition of egg yolk and albumen was determined using inductively coupled plasma optical emission spectrometry (ICP‐OES) following wet acid digestion. Yolk and albumen were separated, homogenized, and freeze‐dried to constant weight prior to analysis. Approximately 0.5 g of dried sample was digested using concentrated nitric acid (HNO_3_) and hydrogen peroxide (H_2_O_2_) under controlled heating until complete mineralization was achieved. Digested solutions were diluted to a fixed volume with ultrapure deionized water. Macro‐elements (Ca, P, Mg, Na, and K) and trace elements (Fe, Zn, Cu, Mn, and Se) were quantified using an ICP‐OES system (Optima 8000, PerkinElmer, USA) calibrated with multi‐element standard solutions. Analytical quality control included reagent blanks, duplicate digestion, and analysis of certified reference materials (CRMs) selected to approximate biological matrices. Instrument performance and calibration stability were verified daily, and only measurements meeting quality‐control acceptance criteria were retained. Because selenium occurs at low concentrations in eggs, particular attention was given to background correction and signal stability. Selenium values approaching the analytical detection limit were interpreted conservatively, and conclusions regarding selenium differences among genotypes were restricted to descriptive or trend‐level interpretation. Mineral concentrations are reported as mg/100 g dry matter. In addition, whole‐egg mineral yields (mg/egg) were calculated by integrating mineral concentrations with egg component masses to enhance nutritional relevance and facilitate comparison among genotypes differing in yolk‐to‐albumen proportion. All mineral determinations were performed on individual eggs, and the individual egg constituted the biological replicate, with analytical duplicates averaged prior to statistical analysis (Altun et al. 2023; Voica et al. 2023).

### 2.6 Statistical Analysis

Data were analyzed using linear mixed‐effects models. Genotypes were included as a fixed effect. Hen identity was included as a random effect to account for repeated egg sampling, and laying day was included to account for short‐term temporal variation. Because each genotype was housed in a single, non‐replicated section, housing section effects could not be statistically separated from genotype effects; accordingly, the results were interpreted as genotype‐associated differences under the present experimental configuration rather than as definitive genetic effects. For morphometric traits, individual eggs were treated as repeated observations. For chemical analyses, the individual egg was considered the biological replicate, and analytical duplicates were averaged prior to statistical analysis. The exact sample size (n) for each analytical endpoint is reported in the corresponding tables. Fatty acid proportions were analyzed as compositional data using centered log‐ratio (CLR) transformation. Statistical inference was performed on CLR‐transformed values, and back‐transformed estimated marginal means are presented. Fatty acid class totals and ratios were calculated arithmetically. Results are reported as estimated marginal means (EMMs) with 95% confidence intervals. Multiple testing was controlled using the Benjamini–Hochberg false discovery rate procedure within each analytical family, with significance accepted at *q* < 0.05. All analyses were performed using IBM SPSS Statistics (v29.0), and figures were generated using GraphPad Prism (v10.6).

### 2.7 Ethical Approval and Animal Welfare

This study was conducted in accordance with institutional guidelines for animal care and welfare. The experimental protocol was reviewed and approved by the Ethics Committee of the Faculty of Natural and Life Sciences, Abdelhamid Ibn Badis University of Mostaganem, Algeria (Approval No. EC‐FSNV‐UABM‐2024‐23). The study involved the collection and analysis of eggs from existing laying hens maintained under routine farm management conditions. No invasive procedure, experimental surgery, or animal sacrifice was performed.

## 3 Results and Discussion

### 3.1 Egg Morphometric Characteristics

Egg morphometric and internal quality traits showed significant differences among Fayoumi, Naked Neck, and Frizzled hens (Table 2). Naked Neck hens produced the heaviest eggs (55.29 g), followed by Frizzled (50.18 g) and Fayoumi hens (48.92 g). The same ranking was observed for egg length and width, with Naked Neck eggs showing the greatest dimensions and Fayoumi eggs the smallest. Albumen and yolk weights were also significantly higher in Naked Neck eggs (34.02 g and 16.25 g, respectively), whereas Fayoumi eggs showed the lowest values (30.45 g and 13.94 g, respectively), and Frizzled eggs displayed intermediate values. Shell weight was highest in Naked Neck eggs (5.02 g), while Fayoumi and Frizzled eggs showed lower values (Table 2).

**TABLE 2:** Egg morphometric and internal quality traits of Fayoumi, Naked Neck, and Frizzled laying hens.

Parameter	Fayoumi	Naked Neck	Frizzled
Egg weight (g)	48.92 ± 3.85ᶜ	55.29 ± 4.11ᵃ	50.18 ± 3.71ᵇ
Egg length (mm)	49.61 ± 0.30ᶜ	53.30 ± 0.28ᵃ	50.10 ± 0.11ᵇ
Egg width (mm)	36.83 ± 0.20ᵇ	38.30 ± 0.18ᵃ	32.20 ± 0.14ᶜ
Albumen weight (g)	30.45 ± 0.95ᶜ	34.02 ± 0.97ᵃ	31.88 ± 1.03ᵇ
Yolk weight (g)	13.94 ± 0.40ᶜ	16.25 ± 0.50ᵃ	13.46 ± 0.32ᵇ
Shell weight (g)	4.95 ± 0.12ᵇ	5.02 ± 0.11ᵃ	4.84 ± 0.10ᵇ
Albumen height (mm)	4.85 ± 0.15ᶜ	5.81 ± 0.14ᵃ	5.13 ± 0.10ᵇ
Haugh unit	76.21 ± 2.10ᶜ	83.39 ± 1.55ᵃ	79.09 ± 2.10ᵇ
Shape index (%)	74.21 ± 4.80ᵃ	71.86 ± 4.11ᵃ	64.27 ± 5.00ᵇ

*Note:* Values are expressed as mean ± SD. Different superscript letters within the same row indicate significant differences among genotypes at *q* < 0.05. *q*‐values correspond to Benjamini–Hochberg false discovery rate‐adjusted *p*‐values. The individual egg was considered the experimental unit for morphometric measurements. *n* = 100 eggs per genotype.

Internal quality indicators followed a similar pattern. Albumen height was highest in Naked Neck eggs (5.81 mm), intermediate in Frizzled eggs (5.13 mm), and lowest in Fayoumi eggs (4.85 mm). Haugh unit values showed the same trend, with the highest value in Naked Neck eggs (83.39), followed by Frizzled (79.09) and Fayoumi eggs (76.21). The shape index also differed among genotypes, with Fayoumi and Naked Neck eggs showing higher values than Frizzled eggs (Table 2). Overall, these results indicate that Naked Neck hens were associated with larger eggs and superior internal quality traits under the present experimental conditions.

### 3.2 Amino Acid Composition

Egg protein content and amino acid composition showed genotype‐associated variation among Fayoumi, Naked Neck, and Frizzled eggs (Table 3). Total protein content ranged from 12.80 g/100 g edible portion in Fayoumi eggs to 13.14 g/100 g in Naked Neck eggs and 13.28 g/100 g in Frizzled eggs, indicating slightly higher protein levels in Naked Neck and Frizzled eggs than in Fayoumi eggs. Despite this relatively narrow range in total protein content, several individual amino acids differed markedly among genotypes.

**TABLE 3:** Protein content and amino acid composition of eggs from Fayoumi, Naked Neck, and Frizzled laying hens.

Parameter	Fayoumi	Naked Neck	Frizzled
Protein (g/100 g edible portion)	12.80 ± 0.60ᵇ	13.14 ± 0.84ᵃ	13.28 ± 0.11ᵃ
Essential amino acids (mg/100 g dry matter)			
Isoleucine	540.10 ± 11.20ᵃ	536.92 ± 13.36ᵃ	540.75 ± 12.00ᵃ
Leucine	610.50 ± 25.30ᵇ	636.27 ± 82.93ᵃ	600.64 ± 18.22ᶜ
Lysine	630.20 ± 18.40ᵇ	615.60 ± 7.02ᵇ	620.00 ± 7.67ᵇ
Methionine	320.40 ± 20.10ᵃ	309.81 ± 5.33ᵇ	271.00 ± 22.00ᶜ
Phenylalanine	510.10 ± 5.20ᵃ	513.53 ± 2.69ᵃ	504.00 ± 6.91ᵇ
Threonine	560.10 ± 20.30ᵇ	506.55 ± 3.03ᵇ	512.44 ± 17.00ᵇ
Valine	650.30 ± 18.60ᵃ	603.11 ± 3.31ᵇ	644.13 ± 4.22ᵃ
Histidine	180.40 ± 10.20ᵇ	110.91 ± 12.65ᶜ	211.55 ± 6.98ᵃ
Non‐essential amino acids (mg/100 g dry matter)			
Aspartic acid	860.30 ± 12.10ᵇ	802.34 ± 11.86ᶜ	833.44 ± 11.00ᵇ
Glutamic acid	800.20 ± 15.30ᵇ	809.63 ± 14.13ᵃ	789.44 ± 13.00ᶜ
Alanine	600.40 ± 10.60ᵃ	540.26 ± 16.30ᶜ	567.80 ± 7.11ᵇ
Arginine	690.50 ± 12.10ᵃ	638.70 ± 4.11ᵇ	635.05 ± 10.08ᵇ
Cysteine	160.20 ± 5.10ᵇ	154.15 ± 3.76ᵇ	171.21 ± 6.88ᵃ
Glycine	450.10 ± 15.30ᵃ	392.13 ± 26.20ᵇ	401.22 ± 5.11ᵇ
Proline	440.20 ± 14.10ᵇ	470.86 ± 1.50ᵃ	395.60 ± 15.00ᶜ
Serine	680.10 ± 6.20ᵃ	676.06 ± 3.89ᵃ	688.90 ± 6.10ᵃ
Tyrosine	255.30 ± 4.60ᵇ	244.62 ± 3.65ᵇ	267.22 ± 5.12ᵃ
EAAs/NEAAs	0.83	0.83	0.84
Predicted PER (P‐PER)	268.5 ± 6.4ᵇ	262.0 ± 5.8ᵇ	244.0 ± 6.2ᶜ

*Note:* Values are expressed as mean ± SD. Different superscript letters within the same row indicate significant differences among genotypes at *q* < 0.05. *q*‐values correspond to Benjamini–Hochberg false discovery rate‐adjusted *p*‐values. Protein content is expressed as g/100 g edible portion, whereas individual amino acids are expressed as mg/100 g dry matter. The individual egg was considered the experimental unit for amino acid analysis.

Among essential amino acids, leucine content was highest in Naked Neck eggs (636.27 mg/100 g dry matter), compared with 610.50 mg/100 g in Fayoumi eggs and 600.64 ± 18.22 mg/100 g in Frizzled eggs. In contrast, methionine concentration was highest in Fayoumi eggs (320.40 mg/100 g), followed by Naked Neck (309.81 mg/100 g) and Frizzled eggs (271.00 mg/100 g). Valine also showed clear genotype‐associated variation, with higher concentrations in Fayoumi (650.30 mg/100 g) and Frizzled eggs (644.13 mg/100 g) than in Naked Neck eggs (603.11 mg/100 g). Histidine displayed the most pronounced difference among the essential amino acids, reaching 211.55 mg/100 g in Frizzled eggs, compared with 180.40 mg/100 g in Fayoumi eggs and only 110.91 mg/100 g in Naked Neck eggs. By contrast, isoleucine remained very stable across the three genotypes, with values close to 537–541 mg/100 g, while lysine and threonine showed more limited variation (Table 3).

Non‐essential amino acids represented the largest fraction of the amino acid pool in all genotypes. Aspartic acid was highest in Fayoumi eggs (860.30 mg/100 g), followed by Frizzled (833.44 mg/100 g) and Naked Neck eggs (802.34 mg/100 g). Glutamic acid, another major non‐essential amino acid, was highest in Naked Neck eggs (809.63 mg/100 g), compared with 800.20 mg/100 g in Fayoumi eggs and 789.44 mg/100 g in Frizzled eggs. Fayoumi eggs also showed the highest alanine (600.40 mg/100 g), arginine (690.50 mg/100 g), and glycine (450.10 mg/100 g) contents, whereas Naked Neck eggs were distinguished by the highest proline level (470.86 mg/100 g). Frizzled eggs were characterized by the highest concentrations of cysteine (171.21 ± 6.88 mg/100 g), serine (688.90 mg/100 g), and tyrosine (267.22 mg/100 g) (Table 3).

The EAAs/NEAAs ratio remained remarkably stable among genotypes, varying only from 0.83 in Fayoumi and Naked Neck eggs to 0.84 in Frizzled eggs, which indicates that genotype‐related differences were driven more by shifts in individual amino acid deposition than by major changes in the overall balance between essential and non‐essential fractions. In contrast, the predicted protein efficiency ratio (P‐PER) differed significantly among genotypes, with higher values in Fayoumi (268.5) and Naked Neck eggs (262.0) than in Frizzled eggs (244.0).

### 3.3 Fatty Acid Composition

Egg yolk fatty acid composition showed clear genotype‐associated variation among Fayoumi, Naked Neck, and Frizzled eggs (Table 4). Total lipid content was highest in Fayoumi eggs (8.92%), followed by Frizzled (7.88%) and Naked Neck eggs (7.36%). Among saturated fatty acids, palmitic acid (C16:0) was the predominant component in all genotypes, ranging from 24.10% in Fayoumi eggs to 25.48% in Frizzled eggs. Total SFA content was slightly but significantly higher in Naked Neck (34.27%) and Frizzled eggs (34.01%) than in Fayoumi eggs (33.10%).

**TABLE 4:** Yolk fatty acid composition of eggs from Fayoumi, Naked Neck, and Frizzled laying hens.

Fatty acid/parameter	Fayoumi	Naked Neck	Frizzled
Total lipids (%)	8.92 ± 0.64ᵃ	7.36 ± 0.61ᵇ	7.88 ± 0.70ᵇ
C14:0	0.18 ± 0.03ᵃ	N.D.	0.07 ± 0.00ᵇ
C15:0	0.03 ± 0.01ᵃ	0.02 ± 0.01ᵇ	N.D.
C16:0	24.10 ± 0.90ᵇ	24.51 ± 0.71ᵇ	25.48 ± 1.80ᵃ
C18:0	8.20 ± 0.45ᵃ	7.99 ± 0.30ᵇ	8.02 ± 0.20ᵇ
C20:0	0.22 ± 0.04ᵃ	0.21 ± 0.03ᵇ	0.20 ± 0.08ᵇ
C22:0	0.13 ± 0.02ᵃ	0.10 ± 0.02ᵇ	0.11 ± 0.01ᵇ
C14:1	0.10 ± 0.02ᵇ	0.18 ± 0.02ᵃ	0.11 ± 0.02ᵇ
C16:1	2.15 ± 0.38ᵇ	2.07 ± 0.45ᵇ	2.32 ± 0.80ᵃ
C18:1 n‐9	44.10 ± 1.10ᵇ	45.04 ± 1.38ᵃ	44.70 ± 1.60ᵃ
C20:1 n‐9	0.20 ± 0.03ᵇ	0.24 ± 0.03ᵃ	0.23 ± 0.04ᵃ
Σ ω6	15.90 ± 0.70ᵇ	17.23 ± 0.50ᵃ	12.12 ± 0.07ᶜ
C18:2 n‐6	15.10 ± 0.85ᵃ	12.97 ± 0.79ᵇ	15.60 ± 0.01ᵃ
C18:3 n‐6	0.04 ± 0.01ᵃ	0.01 ± 0.003ᵇ	0.01 ± 0.001ᵇ
C20:3 n‐6	0.48 ± 0.10ᵇ	0.53 ± 0.17ᵃ	0.58 ± 0.10ᵃ
Σ ω3	2.85 ± 0.20ᵇ	3.62 ± 0.16ᵃ	3.22 ± 0.70ᵇ
C18:3 n‐3	0.62 ± 0.06ᵇ	0.10 ± 0.02ᶜ	0.50 ± 0.02ᵃ
C20:5 n‐3 (EPA)	0.11 ± 0.02ᵇ	0.13 ± 0.02ᵃ	0.11 ± 0.01ᵇ
C22:6 n‐3 (DHA)	1.12 ± 0.08ᵇ	1.26 ± 0.05ᵃ	1.15 ± 0.01ᵇ
SFA	33.10 ± 0.80ᵇ	34.27 ± 0.40ᵃ	34.01 ± 0.22ᵃ
MUFA	47.80 ± 1.10ᵇ	49.61 ± 1.55ᵃ	47.53 ± 0.44ᵇ
PUFA	19.10 ± 0.95ᵃ	16.12 ± 0.10ᶜ	18.46 ± 0.10ᵇ
ω6/ω3 ratio	5.58 ± 1.20ᵃ	4.76 ± 1.40ᵇ	3.76 ± 1.48ᶜ

*Note:* Values are expressed as mean ± SD and reported as relative proportions (% of total identified fatty acids), except for total lipids, which are expressed as % of yolk mass. Different superscript letters within the same row indicate significant differences among genotypes at *q* < 0.05. *q*‐values correspond to Benjamini–Hochberg false discovery rate‐adjusted *p*‐values. The individual egg was considered the experimental unit for fatty acid analysis.

Abbreviation: N.D. = not detected.

Monounsaturated fatty acids also differed significantly among genotypes. Naked Neck eggs exhibited the highest total MUFA proportion (49.61%), whereas Fayoumi and Frizzled eggs showed lower and comparable values (47.80% and 47.53%, respectively). This pattern was mainly associated with oleic acid (C18:1 n‐9), which reached its highest level in Naked Neck eggs (45.04%) compared with 44.10% in Fayoumi eggs and 44.70% in Frizzled eggs. Minor MUFA, including C14:1 and C20:1 n‐9, were also higher in Naked Neck eggs than in the other genotypes (Table 4).

In contrast, polyunsaturated fatty acids displayed a different distribution pattern. Total PUFA content was highest in Fayoumi eggs (19.10%), intermediate in Frizzled eggs (18.46% ± 0.10%), and lowest in Naked Neck eggs (16.12%). Linoleic acid (C18:2 n‐6) represented the major PUFA in all groups and was markedly higher in Frizzled (15.60%) and Fayoumi eggs (15.10%) than in Naked Neck eggs (12.97%). With respect to fatty acid families, total ω6 content was highest in Naked Neck eggs (17.23%), followed by Fayoumi (15.90%) and Frizzled eggs (12.12%), whereas total ω3 content was also highest in Naked Neck eggs (3.62%) compared with 3.22% in Frizzled eggs and 2.85% in Fayoumi eggs.

Within the ω3 series, DHA (C22:6 n‐3) was the predominant long‐chain ω3 fatty acid and reached its highest proportion in Naked Neck eggs (1.26% ± 0.05%), followed by Frizzled (1.15%) and Fayoumi eggs (1.12%). EPA (C20:5 n‐3) was also slightly higher in Naked Neck eggs (0.13%) than in Fayoumi and Frizzled eggs (0.11% and 0.11%, respectively). By contrast, α‐linolenic acid (C18:3 n‐3) was highest in Fayoumi (0.62%) and Frizzled eggs (0.50%) and markedly lower in Naked Neck eggs (0.10%). As a result, the ω6/ω3 ratio differed significantly among genotypes, with the highest value recorded in Fayoumi eggs (5.58), an intermediate value in Naked Neck eggs (4.76), and the lowest ratio in Frizzled eggs (3.76).

### 3.4 Mineral Composition

Egg mineral composition showed selective genotype‐associated variation among Fayoumi, Naked Neck, and Frizzled eggs (Table 5). Sodium content did not differ significantly among genotypes, with values ranging from 114.2 mg/egg in Fayoumi eggs to 116.0 mg/egg in Frizzled eggs. Calcium and phosphorus contents were also comparable across groups, with calcium varying from 43.8 to 46.0 mg/egg and phosphorus from 166.0 to 173.0 mg/egg. Similarly, potassium content showed no significant genotype‐associated difference, with values ranging from 114.8 mg/egg in Fayoumi eggs to 117.0 mg/egg in Naked Neck eggs.

**TABLE 5:** Mineral composition of eggs from Fayoumi, Naked Neck, and Frizzled hens.

Minerals (mg/egg)	Fayoumi	Naked Neck	Frizzled
Sodium (Na)	114.2 ± 10.5ᵃ	115.0 ± 12.8ᵃ	116.0 ± 9.2ᵃ
Calcium (Ca)	45.1 ± 6.2ᵃ	46.0 ± 6.7ᵃ	43.8 ± 5.1ᵃ
Phosphorus (P)	168.4 ± 6.9ᵃ	173.0 ± 13.4ᵃ	166.0 ± 5.1ᵃ
Iron (Fe)	1.24 ± 0.15ᵇ	1.30 ± 0.19ᵃ	1.10 ± 0.08ᶜ
Potassium (K)	114.8 ± 10.1ᵃ	117.0 ± 12.0ᵃ	116.0 ± 10.5ᵃ

*Note:* Values are expressed as mean ± SD and reported as mg/egg. Different superscript letters within the same row indicate significant differences among genotypes at *q* < 0.05. *q*‐values correspond to Benjamini–Hochberg false discovery rate‐adjusted *p*‐values. The individual egg was considered the experimental unit for mineral analysis.

In contrast, iron was the only mineral that differed significantly among genotypes. Naked Neck eggs showed the highest iron concentration (1.30 mg/egg), followed by Fayoumi eggs (1.24 mg/egg), whereas Frizzled eggs exhibited the lowest value (1.10 mg/egg). Overall, these results indicate that major macro‐minerals remained relatively stable across genotypes, whereas iron deposition displayed clearer genotype‐associated variation under the present experimental conditions (Table 5).

### 3.5 Multivariate Analysis of Egg Quality Traits

**FIGURE 1:**
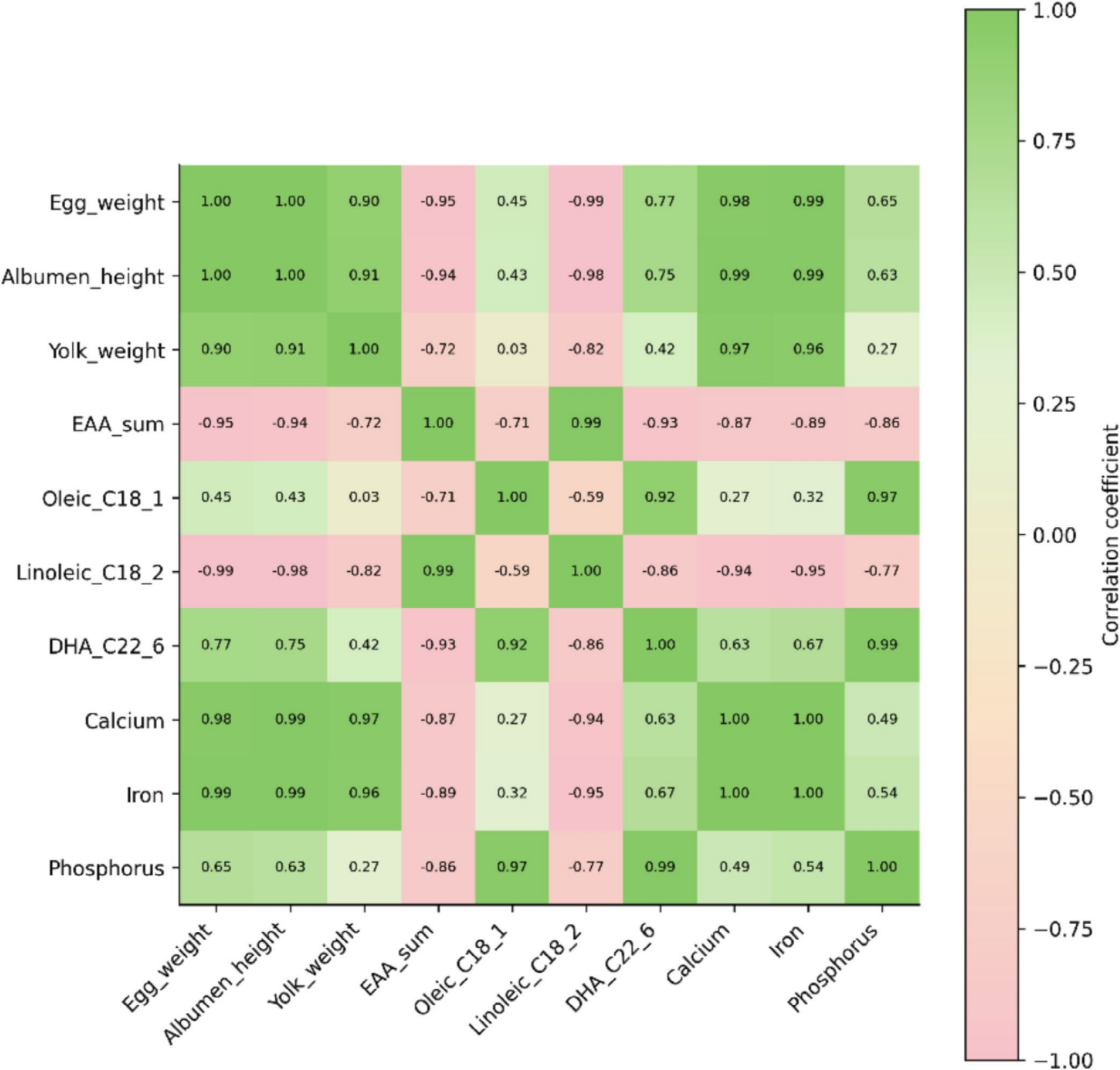
Pearson correlation heatmap of egg morphometric traits, amino acid composition, fatty acid profile, and mineral content.

**FIGURE 2:**
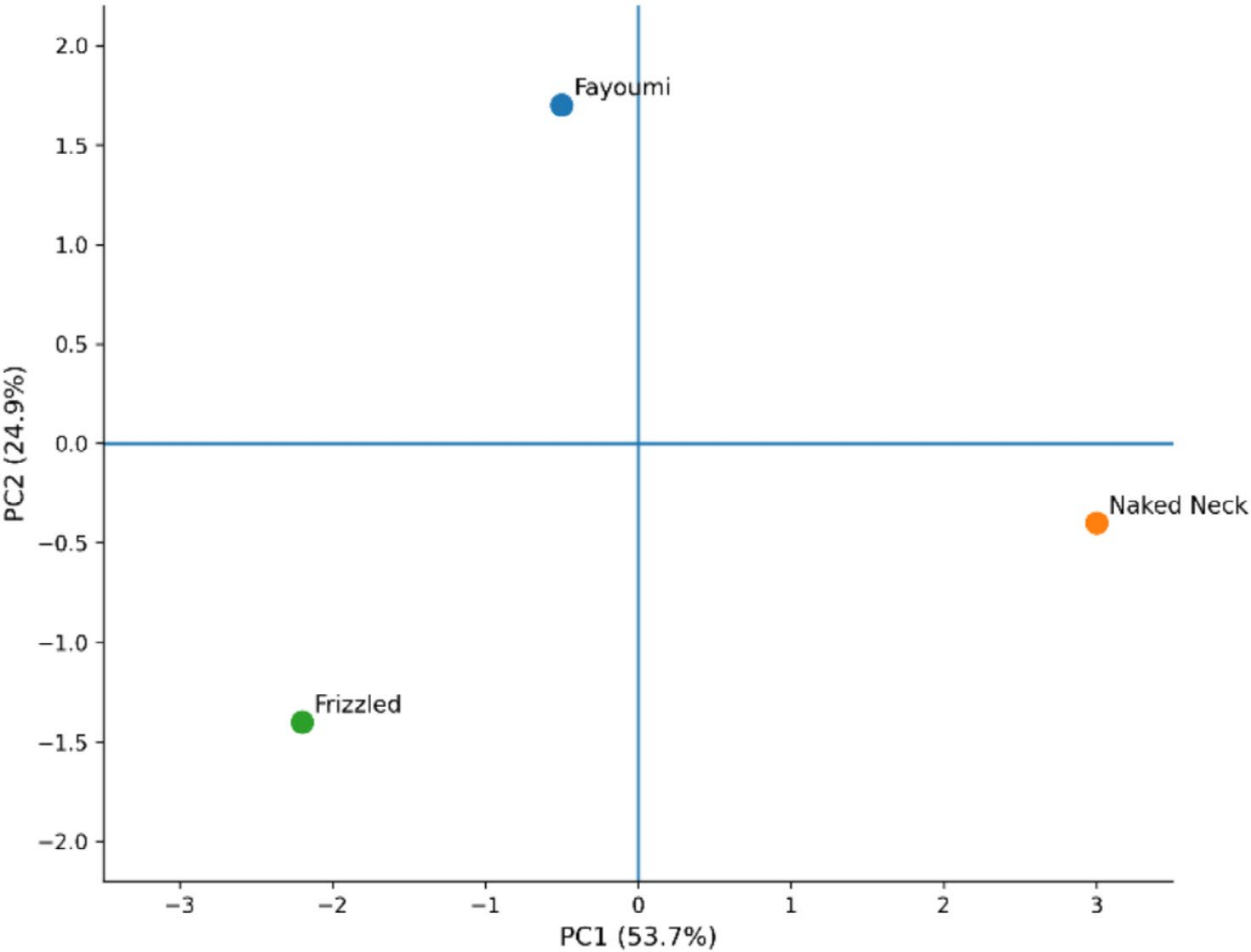
Principal component analysis (PCA) score plot of egg quality, amino acid, fatty acid, and mineral characteristics in Fayoumi, Naked Neck, and Frizzled genotypes.

Pearson correlation analysis and principal component analysis (PCA) provided a complementary overview of the relationships among egg morphometric traits, amino acid composition, fatty acid profile, and mineral content (Figures 1 and 2). The correlation heatmap revealed a structured association pattern in which egg weight, albumen height, and yolk weight were strongly and positively correlated with one another (*r* = 0.90–1.00), indicating coordinated deposition of the main egg components. These morphometric variables were also positively associated with calcium and iron (*r* = 0.96–0.99), suggesting that mineral accumulation was closely linked to egg size and structural development. In contrast, the EAA sum showed strong negative correlations with morphometric traits (*r* = −0.72 to −0.95) and with minerals such as calcium and iron (*r* = −0.87 to −0.89), indicating that variation in amino acid density followed a pattern distinct from that of egg mass‐related traits. Linoleic acid (C18:2 n‐6) showed a similar tendency, being negatively correlated with egg size variables (*r* = −0.82 to −0.99) but positively associated with the EAA sum (*r* = 0.99). By contrast, oleic acid (C18:1) and DHA (C22:6 n‐3) were positively associated with several morphometric and mineral traits, while phosphorus showed particularly strong correlations with oleic acid (*r* = 0.97) and DHA (*r* = 0.99), highlighting coordinated lipid–mineral relationships within the egg matrix.

These correlation patterns were consistent with the PCA distribution. PC1 explained 53.7% of the total variance and PC2 explained 24.9%, giving a cumulative variance of 78.6% (Figure 2). The score plot showed a clear genotype‐associated distribution of samples along the principal axes. Naked Neck eggs were positioned on the positive side of PC1, reflecting their association with traits related to higher lipid and mineral values. Frizzled eggs were located on the negative side of both PC1 and PC2, indicating lower overall contributions from these variables, whereas Fayoumi eggs occupied an intermediate position with a positive loading on PC2, suggesting a relative association with amino acid‐related variation.

## 4 Discussion

The present study showed that Fayoumi, Naked Neck, and Frizzled hens produced eggs with distinct morphometric, amino acid, fatty acid, and mineral profiles under summer hot‐climate conditions. Since each genotype was maintained in a separate non‐replicated housing section, the findings should be interpreted as genotype‐associated patterns observed under the present production setting rather than as definitive genetic effects. This cautious interpretation is important because egg quality and composition are influenced by genetic background, environmental conditions, diet, and management, even when the main production conditions are standardized (Franco et al. 2020; Fernandes et al. 2023; Hejdysz et al. 2024; Gyawali and Goto 2025).

Naked Neck hens produced heavier eggs and showed better internal quality indicators than Fayoumi and Frizzled hens. This pattern suggests greater albumen and yolk deposition, together with better maintenance of albumen quality under hot‐climate conditions. The response is biologically plausible because the Naked Neck genotype is characterized by reduced feather coverage, which facilitates sensible heat loss and may reduce the physiological cost of thermoregulation during thermal stress (Akbarian et al. 2016; Attia et al. 2018; Fernandes et al. 2023; Hemanth et al. 2024). Improved heat dissipation may allow a greater proportion of metabolic resources to be directed toward egg formation, particularly albumen deposition and internal quality maintenance. Fayoumi eggs were generally smaller, whereas Frizzled eggs showed an intermediate morphometric profile, indicating that thermotolerant genotypes may differ in their allocation of nutrients toward egg size and structural quality (Fathi et al. 2022; Nawaz et al. 2024; Adomako and Asamoah 2025; Juárez et al. 2025).

Amino acid composition varied among genotypes, although total protein content remained relatively close across groups. Fayoumi eggs were characterized by higher methionine, Naked Neck eggs by higher leucine, and Frizzled eggs by higher histidine. These differences suggest that genotype‐associated variation was driven mainly by changes in individual amino acid deposition rather than by major differences in total protein accumulation. This distinction is nutritionally relevant because sulfur‐containing amino acids, branched‐chain amino acids, and histidine contribute differently to protein quality, tissue metabolism, antioxidant defense, and physiological function (Alagawany et al. 2021; Goto et al. 2021; Gyawali and Goto 2025). The relatively stable EAAs/NEAAs ratio further indicates that the global amino acid balance was preserved, while specific amino acids showed genotype‐related enrichment, which agrees with previous evidence that breed and genetic background can influence egg amino acid traits without necessarily producing large differences in total protein content (Ariza et al. 2021; Goto et al. 2021; Gyawali and Goto 2025).

The fatty acid profile represented one of the clearest sources of differentiation among the evaluated eggs. Fayoumi eggs showed higher total lipid and PUFA proportions, Naked Neck eggs were distinguished by higher MUFA and total ω3 levels, including DHA, whereas Frizzled eggs showed the lowest ω6/ω3 ratio. These findings indicate that thermotolerant genotypes may differ not only in the quantity of yolk lipid deposition but also in the qualitative organization of the lipid fraction. This interpretation is consistent with previous studies showing that hen genotype can modulate egg lipid composition, fatty acid distribution, and nutritionally relevant lipid indices under comparable environmental or dietary conditions (Cartoni Mancinelli et al. 2022; Hejdysz et al. 2024; Oketch and Heo 2025). From a nutritional point of view, these differences are important because egg lipids contribute to dietary fatty acid intake, and eggs may represent an accessible source of long‐chain ω3 fatty acids in human diets (Cartoni Mancinelli et al. 2022; Caffa et al. 2025). However, these fatty acid results should be interpreted as relative compositional profiles, since values were expressed as percentages of total identified fatty acids rather than absolute concentrations.

Mineral composition was more conserved than amino acid and fatty acid composition. Sodium, calcium, phosphorus, and potassium remained relatively stable across genotypes, suggesting tight physiological regulation of major mineral deposition in eggs. This stability is expected because major minerals are closely involved in osmotic balance, shell formation, and egg structural integrity, which are strongly regulated by reproductive and mineral metabolism (Hincke et al. 2019; Altun et al. 2023; Voica et al. 2023). In contrast, iron showed clearer genotype‐associated variation, with the highest value observed in Naked Neck eggs. This selective difference suggests that trace mineral deposition may be more sensitive to genotype‐related physiological or metabolic differences than macro‐mineral deposition. Although the magnitude of mineral variation was smaller than that observed for lipid and amino acid traits, it remains nutritionally meaningful because eggs contribute to the intake of bioavailable micronutrients, particularly in diets with limited access to diversified animal‐source foods (Akinwumi et al. 2025; Caffa et al. 2025).

Overall, the results indicate that each genotype was associated with a specific egg quality and nutritional profile. Naked Neck eggs combined greater egg mass, better internal quality, and a more pronounced MUFA/ω3 profile. Fayoumi eggs were characterized by higher total lipid and PUFA proportions, whereas Frizzled eggs showed intermediate morphometric traits together with the lowest ω6/ω3 ratio. These patterns suggest that thermotolerant chicken genotypes may offer complementary advantages for egg production under hot‐climate conditions, depending on whether the priority is egg size, internal quality, lipid profile, or fatty acid balance (Kennedy et al. 2022; Fernandes et al. 2023; Olaniyan et al. 2024; Dessie et al. 2025).

The main limitation of this study is the absence of replicated housing sections, which prevents complete separation of genotype effects from possible section‐related environmental influences. In addition, sulfur‐containing amino acids should be interpreted with caution because performic acid oxidation was not applied before hydrolysis, and fatty acid data should be considered relative rather than absolute. Despite these limitations, the study provides an integrated comparison of physical and nutritional egg quality in three thermotolerant genotypes maintained under the same general farm conditions. These findings support the nutritional valorization of thermotolerant poultry genetic resources and highlight the need for replicated experimental studies to confirm how genotype shapes egg composition under hot‐climate production systems (Fernandes et al. 2023; Hejdysz et al. 2024; Gyawali and Goto 2025; Caffa et al. 2025).

## 5 Conclusion

The present study demonstrated that eggs produced by Fayoumi, Naked Neck, and Frizzled hens differed in both physical quality and nutritional composition under summer hot‐climate conditions. Clear variation was observed in egg morphometric traits, internal quality indices, amino acid composition, yolk fatty acid profile, and iron content, indicating that the evaluated genotypes were associated with distinct patterns of nutrient deposition within the egg matrix. Among the three groups, Naked Neck eggs were characterized by greater egg mass, higher albumen height, and higher Haugh unit values, together with a more pronounced MUFA and long‐chain ω3 profile. Fayoumi eggs, in contrast, showed higher total lipid deposition and higher overall PUFA representation, whereas Frizzled eggs displayed generally intermediate morphometric characteristics combined with the most favorable ω6/ω3 ratio. Differences in individual amino acids and iron concentration further supported the existence of compositional distinctions among the evaluated eggs.

From a nutritional perspective, these findings highlight the potential value of thermotolerant chicken resources as suppliers of eggs with differentiated compositional profiles in climate‐constrained production systems. The results further suggest that, beyond egg size alone, the qualitative distribution of proteins, lipids, and minerals within the egg can vary among thermotolerant genotypes reared under the same general farm conditions. This is particularly relevant in hot environments, where identifying poultry resources that combine adaptation capacity with satisfactory egg quality remains important for both production sustainability and nutritional valorization.

## Author Contributions

**Fatiha Soltani:** conceptualization, methodology, writing – original draft. **Golam Sagir Ahammad:** writing – review and editing, supervision, data curation, conceptualization. **Djilali Benabdelmoumene:** writing – review and editing, supervision, software, methodology, investigation. **Wasim S. M. Qadi:** investigation, validation, methodology, software, supervision. **Muhammad Waqar:** supervision, project administration, visualization, writing – review and editing, conceptualization. **Zineb Bengharbi:** conceptualization, investigation, validation, formal analysis. **Said Dahmouni:** visualization, project administration, resources, data curation. **Ahmed mediani:** resources, writing – review and editing, investigation, supervision.

## Funding

The authors have nothing to report.

## Conflicts of Interest

The authors declare no conflicts of interest.

## Data Availability

The data that support the findings of this study are available from the corresponding author upon reasonable request.
